# Quality of life in children and adolescents with allergic rhinitis

**DOI:** 10.1016/S1808-8694(15)30511-5

**Published:** 2015-10-18

**Authors:** Carlos Henrique Martins da Silva, Taís Estevão da Silva, Nívea Macedo O. Morales, Karla P. Fernandes, Rogério M.C. Pinto

**Affiliations:** 1 Doctor, professor of the Pediatrics Department, Faculdade de Medicina da Universidade Federal de Uberlândia; 2 Resident in pediatrics (second year resident in pediatrics), Universidade Federal de Uberlândia; 3 Doctor, neuropediatrician of the Hospital de Clínicas da Faculdade de Medicina da Universidade Federal de Uberlândia and the Associação de Assistência a Criança Deficiente, AACD; 4 Master's degree, in charge of the Child Allergology Unit of the Hospital de Clínicas da Faculdade de Medicina da Universidade Federal de Uberlândia; 5 Doctor, professor at the Faculdade de Matemática da Universidade Federal de Uberlândia

**Keywords:** adolescents, children, quality of life, rhinitis

## Abstract

Allergic rhinitis (AR) remains a significant pediatric health problem because of the burden of uncontrolled symptoms on daily activities and on general well being.

**Aim:**

to assess the impact of AR on health-related quality of life (HRQL) of children and adolescents using a generic instrument, the Child Health Questionnaire (CHQ - PF50).

**Methods:**

Between January and November 2004, parents or caregivers of 23 children and adolescents with AR without comorbidities and with positive prick tests for at least one air allergen were invited to participate of a cross-sectional study and asked to answer the self-administered CHQ-PF50. The scores were compared to those of healthy children and adolescents.

**Results:**

Patient scores were lower (p<0.05) than healthy subsets in both the physical and psychosocial summaries and in most of the CHQ-PF50 scales (p<0,05), except for the “change in health” scale. The size effect was higher in the physical score compared to the psychosocial summary score.

**Conclusions:**

allergic rhinitis has a global negative impact on the HRQL of children and adolescents, with major repercussions in physical function; AR also negatively affects family relations.

## INTRODUCTION

Three fundamental reasons have been given for treating patients: to prolong life, to reduce morbidity and to seek well-being.^1^ Until recently, the success of therapy was evaluated only according to morbimortality rates. Measurements based on physiological and laboratory tests described the severity of disease, and well-being was believed to be the natural consequence of reducing such severity.^2^, ^3^, ^4^ However, evidence has shown that objects parameters correlate poorly or moderately with the well-being of patients. A given clinical severity may have diverse effects on the well-being of different patients because of tolerance levels, health expectations and the ability to deal with disease-imposed limitations. Any evaluation should embody the subjective perceptions of patients about their own status and quality of life (QOL).^2^, ^5^, ^6^

The World Health Organization (WHO) defines QOL as “individuals' perception of their position in life in the context of the culture and value systems in which they live and in relation to their goals, expectations, standards and concerns.”^7^ The term “health-related quality of life” (HRQL) derived mostly from a redefinition of health by the WHO in 1948: “a state of full physical, mental and social well-being, and not merely absence of illness or disease.”^8^

HRQL investigation includes features such as subjectivity, multidimensionality (physical, psychological and social domains) and bipolarity (positive and negative domains, such as autonomy and dependence).^7^ Many questionnaires for assessing the HRQL have been tested; they are classified as generic or specific.^2^, ^9^

Generic instruments comprise domains applicable to many diseases and population groups; it is possible to compare diagnoses and healthy × ill groups. Specific instruments assess the HRQL in diseases and/or specific populations; it is more sensitive for detecting change.^9^, ^10^

Allergic rhinitis (AR) is one of the diseases of childhood that affects the HRQL. It is an inflammation of the nasal mucosa due to an IgE-mediated hypersensitivity reaction to specific allergens that occurs in genetically predisposed and sensitized individuals; one or more of the following symptoms may be present: nose block, watery rhinorrhea, sneezing, and nasal itching.^11^

The prevalence of this disease has increased in recent decades partly due to environmental exposure to allergens, altered life styles (more time spent in closed environments), and social and economic factors. World statistics show 30% to 40% prevalence in children and teenagers. Studies in Brazil have shown a 33% and 34% prevalence in school children aged 6-7 years and 13-14 years.^11^ The prevalence was 24% among school-aged children (6-7 years) in the city of Uberlandia.^12^

Although there is a trend to underestimate the repercussions of AR on the QOL of individuals - because it is a benign disease that rarely requires hospitalization, surgery or complex interventions^1^, ^6^ - AR may cause physical, psychological and social changes in patients, which need to be investigated.^2^,^3^,^13^, ^14^, ^15^

Assessments of the impact of AR on the HRQL became more common in the 1990s.^1^, ^4^ The first such studies were carried out in adults, using generic and specific instruments.^16^, ^17^, ^18^, ^19^ There were fewer studies done in children and teenagers, only with specific questionnaires.^20^, ^21^ Nascimento Silva^22^ adapted and validated a specific questionnaire (RQLQ) for Brazilian adolescents with rhinitis. The current recommendation is to include HRQL studies using specific and generic instruments in clinical trials on chronic respiratory diseases, as well as investigation of objective clinical parameters such as pulmonary functions tests (asthma) and symptom evaluation scales (rhinitis).^23^, ^24^

The purpose of this study was to assess the impact of AR on the HRQL of children and teenagers by applying a generic instrument - the Child Health Questionnaire (CHQ-PF50).

## SERIES AND METHOD

A cross-sectional study was undertaken from January to November 2004, following approval by the local Research Ethics Committee (n° 095/2002).

### Participants

Invitations were sent to parents or guardians of children aged over 5 years and teenagers with a diagnosis of AR for at least one year, based a the clinical history of suggestive symptoms (sneezing, rhinorrhea, nasal itching and/or obstruction) in suggestive circumstances (exposure to known allergens) and with positive immediate hypersensitivity skin tests for at least one of the following aeroallergens: Blomia tropicalis, Dermatophagoides pteronyssinus, Dermatophagoides farinae, storage acarid mites, fungi, pollen, feathers, Blatella germanica, wool, dog and cat epithelium.

Patients with other chronic diseases or comorbidities that might have altered the HRQL investigation, such as asthma, atopic eczema, sinusitis, or food allergy, patients using medication that could alter the skin tests, and patients that refused the skin tests were excluded.

A control group was formed from a database of healthy children and teenagers with no history of respiratory allergies, evaluated during validation of the Brazilian version of the CHQ-PF50;21 these children were matched for age and sex in a 3:1 proportion for this study.

### “Child Health Questionnaire - CHQ-PF50”

The “50-item, parent completed short form, Child Health Questionnaire” (CHQ-PF50) is a generic questionnaire for assessing the HRQL, applicable to children aged over 5 years and teenagers, in the perspective of parents or guardians. It was developed in Boston, US, between 1990 and 1996,^25^ and adapted culturally and validated for the Brazilian population.^26^, ^27^ This instrument consists of 50 items distributed in 4 simple scales and 11 multiple item scales; it encompasses 11 child domains and 4 family domains (Table 1). Scores range from 0 to 100; highest scores indicate better functioning or feeling. There are two summary component scores of physical and psychosocial health.^25^

**Table 1:** Interpretation of domain scores of the CHQ PF-50

Domain	Low score	High score
General health assessment	In general, the child's health is poor	In general, the child's health is excellent
Physical functioning*	Child is limited in carrying out all physical activities, including self-care, due to health issues	Child carries out all types of activities, including those that require effort, with no limits due to health issues
Limitation due to emotional aspects*	Child is limited in carrying out schoolwork and activities with friends due to emotional or behavioral issues	Child is not limited by emotional or behavioral issues in schoolwork and activities with friends
Limitation due to the physical function*	Child is very limited in carrying out schoolwork or activities with friends due to physical health issues	Child is not limited by physical health issues in schoolwork or activities with friends
Bodily pain*	Child has severe and frequent pain	Child has no bodily pain
Behavior*	Child often discusses, finds it hard to concentrate, lies, steals, is obstinate or irritated/willful	Child does not discuss, has no concentration problems, has not lied, stolen, become obstinate or irritated/willful
General behavior	Child behavior is poor compared to other children	Child's behavior is excellent compared to other children
Mental health*	Child cries episodically, feels alone, nervous, bothered or annoyed	Child has no episodes of crying, does not feel alone, nervous, bothered or annoyed
Self-esteem*	Child is not satisfied with abilities, appearance, relationships and life in general	Child is satisfied with abilities, appearance, relationships and life in general
Perception of health	Parents believe that their child's health is poor and tends to become worse	Parents believe that their child's health is excellent and will continue to be
Change in health	Child's health is poorer now compared to a year ago	Child's health is better now compared to a year ago
Emotional impact on parents *	Parents are greatly concerned or bothered with the physical or psychosocial health of the child	Parents are not worried or bothered with the physical or emotional health of the child
Time impact on parents*	Parents feel limited in their own needs due to the physical or psychosocial health of the child	Parents do not feel limited in their own needs because of the physical or psychosocial health of the child
Family activities*	The child's health often limit/interrupts family activities or causes distress	The child's health never limits/interrupts family activities or causes distress
Family cohesion	The ability of the family to understand each other is poor	The ability of the family to understand each other is excellent

* Questions refer only to the past four weeks

### Procedure

Parents and guardians signed a free informed consent form adapted to the Portuguese language and answered a protocol with demographic data (sex, age, education level and number of siblings of child, education of informant and family monthly income) and information about the disease (severity and duration). The CHQ-PF50 questionnaire was applied just before or immediately after a consultation, using a self-application technique.

Patients were asked to indicate an overall state of discomfort due to AR on an analog visual scale (AVS) measuring 100 mm with no subdivisions, where 0 was lack of discomfort and 100 was extreme discomfort. Discomfort due to each symptom (nasal block, sneezing, coryza and nasal itching) was scored from 0 to 6, where 0 was absence and 6 was extreme discomfort. Categories are subdivisions of 24 points, as follows: 0 (no discomfort), 1-8 (mild discomfort), 9-16 (moderate-severe discomfort) and 17-24 (severe discomfort).

Patients were classified according to the severity of AR as mild and moderate-severe, according to the consensus criteria of the Allergic Rhinitis and its Impact on Asthma (ARIA).^28^

CHQ-PF50 scores of AR patients were compared with the control group scores and correlated with severity and overall discomfort of AR (AVS) and degree of discomfort due to each symptom.

The reliability of the instrument - verification of measurement errors and accuracy of scales and domains - was established by internal consistency, the degree by which items are interrelated.

### Statistical analysis

Descriptive statistical analysis was applied to characterize social and demographic features of participants.

Internal consistency was measured using the Cronbach α coefficient. Cronbach's α coefficient is based on the number of items in a scale and their homogeneity. For comparisons of groups, 0.5 to 0.7 (or more) minimal reliability measures are recommended.

Student's t test was applied for comparing the scores of AR patients and healthy children and teenagers.

The effect size was applied to assess the magnitude of differences found in each scale and in the summary scores; the control group mean was subtracted from the mean of the study group and the result was divided by the standard deviation of the control group. A 0.20 to 0.49 result was considered small, a 0.50 to 0.79 result was considered moderate, and a result of 0.80 or more was considered large.^29^

The point biserial correlation coefficient was applied to verify correlations among scales and summary scores in the CHQ and the AVS.

Pearson's correlation coefficients (quantitative variables) and Spearman's correlation coefficients (qualitative variables) were applied to verify correlations among scales and summary scores of the CHQ and disease severity, and discomfort categories due to each symptom.

The significance level was 0.05.

## RESULTS

### Participants

There were 44 patients with a diagnosis of AR (without asthma or other comorbidities) in the outpatient unit. Two of these abandoned the study, 6 were aged below 5 years, 4 did not return the questionnaire, 5 had negative skin tests, and 4 did not answer the questionnaire, and were all excluded from the study. At the end there were 23 questionnaires available for analysis. Twenty-one patients had persistent AR, and two patients had intermittent AR.

Informants were mostly mothers (86.95%). The mean age of patients was 9.22 years; most were male (78.26%). Table 2 shows additional features of the sample.

**Table 2:** General characteristics of participants

Characteristics	Participants (n=23)
Mean age in years (range)	9,2 (5-14)
Male sex (%)	18 (78,3%)
Mean education level in years (range)	3,2 (0-7)
Mean number of siblings (range)	1 (0-5)
Severity of rhinitis (ARIA), n (%)	
Mild	5 (21,7%)
Moderate - Severe	18 (78,3%)
Mean duration of disease in years (range)	5,2 (0,7-14)
General degree of discomfort caused by rhinitis (analog visual scale), mean (range)	53 (1,1-100)
Degree of discomfort of main symptoms, n (%)	
Mild	8 (34,8%)
Moderate - severe	12 (52,2%)
Severe	3 (13,0%)

Cronbach's alpha coefficients were above 0.7, except for the perception of health scale (0.3).

Scores in the study group were lower compared to the control group, except for the change in health scale. The difference between groups was not significant only in the scales general behavior and family activities (p=0.108 and p equals; 0.081). The size effect was significant in summary scores and in 10 scales, and moderate in 5 scales; it was higher in the physical summary score (1.83) compared to the psychosocial summary score (0.89) (Table 3).

**Table 3:** CHQ-PF50 scores in patients and controls

Scales and summaries - CHQ-PF50	Controls (n equals; 69)		Patients (n= 23)			
	Mean	Standard deviation	Mean	Standard deviation	p value*	Size effect
General health	93,26	12,03	62,17	25,26	0,000	2,58
Physical functioning	98,47	8,93	88,52	21,78	0,043	1,11
Limitations due to emotional aspects	97,42	8,76	85,85	23,05	0,031	1,32
Limitations due to the physical function	98,06	12,13	86,23	21,70	0,019	0,97
Bodily pain or discomfort	96,96	9,28	70,43	19,88	0,000	2,86
Behavior	78,17	12,52	65,46	19,74	0,007	1,01
General behavior	85,00	16,13	75,65	25,37	0,108	0,58
Mental health	78,95	11,85	70,87	17,17	0,014	0,68
Self-esteem	93,06	13,46	82,44	23,35	0,048	0,79
Perception of health	79,01	11,90	64,00	17	0,000	1,26
Change in health	67,54	23,84	84,78	19,57	0,001	−0,72
Emotional impact on parents	83,70	21,93	63,76	30,42	0,007	0,91
Time impact on parents	93,30	15,85	79,03	27,74	0,030	0,90
Limitations in family activities	89,19	14,26	82,71	17,95	0,081	0,45
Family cohesion	78,48	18,28	61,09	30,78	0,016	0,95
Summary score of physical health	56,65	5,33	46,89	10,53	0,000	1,83
Summary score of psychosocial health	54,03	6,54	48,23	7,72	0,001	0,89

* Student's t test

The analog visual scale was positively correlated and significant in the general behavior scale (r=0.42) and negative and significant in the change in health scale (r= -0.52) (Table 4).

**Table 4:** Correlation among CHQ-PF50 scores and the analog visual scale

Scales and summaries - CHQ-PF50	AVS	
	r	p-value
General health	0,10	0,62
Physical functioning	0,32	0,12
Limitations due to emotional aspects	0,24	0,28
Limitations due to the physical function	0,29	0,17
Bodily pain or discomfort	−0,34	0,11
Behavior	0,05	0,82
General behavior	0,42	0,04
Mental health	0,22	0,29
Self-esteem	0,13	0,54
Perception of health	−0,04	0,84
Change in health	−0,52	0,00
Emotional impact on parents	0,28	0,19
Time impact on parents	0,30	0,16
Limitations in family activities	0,17	0,42
Family cohesion	−0,11	0,60
Summary score of physical health	0,15	0,49
Summary score of psychosocial health	0,09	0,68

AVS equals; analog visual scale

Disease severity and discomfort due to each symptom did not correlate significantly with CHQ scores.

## DISCUSSION

This is the first study to assess the HRQL in children and teenagers with AR with the generic questionnaire CHQ-PF 50.

The internal consistency reliability of the instrument items was adequate except for the perception of health scale, as also observed in the Brazilian version of the CHQ-PF50 for a healthy population, for subjects with juvenile idiopathic arthritis and with cerebral palsy.^27^, ^30^, ^31^ This result may suggest issues with the cultural adaptation of this instrument or difficulty in understanding items of the scale.

The results show that AT has a negative multidimensional impact on the HRQL. The repercussion is more pronounced in the physical health domain compared to the psychosocial domain (larger size effect in the physical summary score); this has been described in other studies of adults and children.^17^, ^18^, ^21^ The lowest score was found in the control group in the change in health scale, as expected for a healthy population with no change in health across time.

Some studies^16^,^20^,^21^,^32^ have documented AR-related situations that caused most discomfort to patients: nasal symptoms, associated symptoms (headache, tearing, increased thirst, weariness/fatigue, concentration difficulty, insomnia); emotions (irritation, frustration due to limits caused by the disease on daily activities, disquiet, impatience, anger, nervousness, shame due to nasal symptoms), and practical problems (repeatedly scratching and blowing the nose, carrying handkerchiefs, using medication).

In children, nasal symptoms and practical matters may bother school colleagues and cause embarrassment and labeling. Schoolwork may be affected due to distraction, fatigue, irritability, side effects of medication, or absenteeism. Therapeutic control measures to avoid contact with allergens may limit recreational activities and contact with colleagues, leading to social isolation.^4^

A Brazilian study of teenagers showed that physical symptoms (mostly nasal), rather than emotional factors, were the most frequently mentioned items as causing discomfort. Other discomfort factors mentioned were weariness, malaise, thirst, anxiety, nervousness, use of medication, and embarrassing situations due to symptoms.^22^

The results of family-related domains indicate that the disease has a negative impact on the family. Studies of chronic diseases in childhood have shown that these conditions may affect the QOL of families as follows: lack of sleep, fatigue, absence from work, cancellation of vacations, interference on social life, and financial costs. Some parents may feel guilt, become anxious or over-protective, or even hostile towards the child, which may have a negative effect on the family group.^4^, ^33^

The correlation between the AVS and the change in health domain suggest that increased discomfort due to AR as perceived by the child is correlated with a poorer perception of improvement in health as seen by the parents, which may be a challenge for successful therapy in patients with greater discomfort.

Non-agreement between general behavior and behavior scales in the correlation with the AVS may be due to a different approach towards the theme; the former scale compares the child's behavior with that of other children in one item only, while the latter includes five situations, questioning the child's behavior in each one. These results suggest that parents do not perceive behavior differences when comparing their children with other children, although they may be able to report a worse behavior in daily situations. Specific instruments evaluating this domain may provide more accurate information.

### Limitation of this study

Although genetic studies of the HRQL make it possible to compare patients with different chronic conditions, they may be indifferent to changes in specific conditions, since their focus is not a specific disease.^9^ This methodological limitation, inherent to generic instruments, may explain the conflicting results we found.

A representative filling in a HRQL questionnaire is a situation that, a priori, conflicts with the underlying principle of seeking an evaluation by the patient him or herself about their status. Children vary in the development of basic understanding of meanings and functions of the words health and disease, which may preclude effective communication. Agreement among answers by children and those of parents or guardians remains controversial, but has been confirmed in some studies.^10^, ^34^

AR often is associated with other allergies, especially asthma. Our study aimed, however, to investigate the impact of AR singly. Such rigor in the inclusion criteria restricted the participation of subjects with other atopic conditions, which was reflected in the sample size.

## CONCLUSION

AR causes a global negative impact on the HRQL of children and teenagers, altering mostly the physical function, according to the perception of parents or guardians, and affects negatively the family group.
